# Examining the relationship between angiopoietin‐2 and cognition in early Alzheimer’s disease

**DOI:** 10.1002/alz.091813

**Published:** 2025-01-09

**Authors:** Bing Xin Song, Erika Beroncal, Ana C. Andreazza, Damien Gallagher, Susan Marzolini, Mark J. Rapoport, Jocelyn Charles, Nathan Herrmann, Paul I. Oh, Alex Kiss, Walter Swardfager, Bradley J. MacIntosh, Tarek K. Rajji, Giovanni Marotta, Mehreen Siddiqui, Danielle Vieira, Raphael Kusumo, Krista L. Lanctôt

**Affiliations:** ^1^ University of Toronto, Toronto, ON Canada; ^2^ Sunnybrook Research Institute, Toronto, ON Canada; ^3^ Temerty Faculty of Medicine, Department of Psychiatry, University of Toronto, Toronto, ON Canada; ^4^ Sunnybrook Health Sciences Centre, Toronto, ON Canada; ^5^ KITE Research Institute, Toronto Rehabilitation Institute, Toronto, ON Canada; ^6^ Centre for Addiction and Mental Health, Toronto, ON Canada

## Abstract

**Background:**

Angiopoietin‐2, which regulates endothelial permeability and angiogenesis functions, is emerging as a potential biomarker for early Alzheimer’s disease (AD). Elevated levels of cerebrospinal fluid angiopoietin‐2 concentrations have been found in early AD and may be associated with the breakdown of blood‐brain barrier that can contribute to cognitive decline. There is a lack of research on blood angiopoietin‐2 levels and cognition in early AD. Hence, we examined the association between serum angiopoietin‐2 and global cognition.

**Method:**

Participants were diagnosed with mild cognitive impairment (MCI) due to AD or mild Alzheimer’s dementia using the Diagnostic and Statistical Manual of Mental Disorders 5 criteria for mild or major neurocognitive disorder. Serum angiopoietin‐2 concentrations (ng/mL) were measured and analyzed using enzyme‐linked immunosorbent assays. Global cognition was assessed by the Montreal Cognitive Assessment (MoCA). Linear regression analyses were used to assess the association between angiopoietin‐2 levels and cognition adjusting for covariates selected *a priori*: age and the presence of cardiovascular risk factors or disease (cardiovascular status). Angiopoietin‐2 levels were log‐transformed prior to analyses due to significant departure from normality in Shapiro‐Wilk tests.

**Result:**

Of 30 participants [17 (57%) male, 21 (70%) MCI, 22 (73%) with cardiovascular risk factors or disease], the mean (SD) age was 74.8 (8.7), years of education was 16.3 (2.2), MoCA total score was 21.9 (3.4), and angiopoietin‐2 levels were 4.0 (3.6) ng/mL. Higher angiopoietin‐2 was associated with better cognition [F(3,26) = 6.7, p = .002, R^2^ = .44]. Controlling for age and cardiovascular status, one log angiopoietin‐2 unit was associated with a 4.2 (SE= 2.0) higher score in global cognition (p = .048, f^2^ = .10).

**Conclusion:**

In this sample, higher serum angiopoietin‐2 concentrations were associated with better global cognition, controlling for age and cardiovascular status. This relationship supports the potential involvement of angiogenesis in cognitive functions and neurodegenerative diseases and suggests the need for further research to explore cardiovascular functions and cognition in early AD.

